# Whole genome analysis of *Bacillus amyloliquefaciens* TA-1, a promising biocontrol agent against *Cercospora arachidicola* pathogen of early leaf spot in *Arachis hypogaea L*

**DOI:** 10.1186/s12870-023-04423-4

**Published:** 2023-09-04

**Authors:** Chen Wang, Taswar Ahsan, Ao Ding, Di Han, Chao-Qun Zang, Yu-Qian Huang, Khalid Hussain

**Affiliations:** 1 Plant Protection College, Shenyang Agricultural University, Shenyang, 110866 China; 2 Institute of Plant Protection, Liaoning Academy of Agricultural Sciences, Shenyang, 110161 P.R. China; 3 Department of Botany, University of Gujrat, 50700 Gujrat, Pakistan

**Keywords:** Peanut early leaf spot, *Cercospora arachidicola*, *Bacillus amyloliquefaciens*, Biocontrol, Secondary metabolites

## Abstract

**Background:**

Early leaf spot disease, caused by *Cercospora arachidicola*, is a devastating peanut disease that has severely impacted peanut production and quality. Chemical fungicides pollute the environment; however, *Bacillus* bacteria can be used as an environmentally friendly alternative to chemical fungicides. To understand the novel bacterial strain and unravel its molecular mechanism, De novo whole-genome sequencing emerges as a rapid and efficient omics approach.

**Results:**

In the current study, we identified an antagonistic strain, *Bacillus amyloliquefaciens* TA-1. In-vitro assay showed that the TA-1 strain was a strong antagonist against *C. arachidicola*, with an inhibition zone of 88.9 mm. In a greenhouse assay, results showed that the TA-1 strain had a significant biocontrol effect of 95% on peanut early leaf spot disease. De novo whole-genome sequencing analysis, shows that strain TA-1 has a single circular chromosome with 4172 protein-coding genes and a 45.91% guanine and cytosine (GC) content. Gene function was annotated using non-redundant proteins from the National Center for Biotechnology Information (NCBI), Swiss-Prot, the Kyoto Encyclopedia of Genes and Genomes (KEGG), clusters of orthologous groups of proteins, gene ontology, pathogen-host interactions, and carbohydrate-active enZYmes. antiSMASH analysis predicted that strain TA-1 can produce the secondary metabolites siderophore, tailcyclized peptide, myxochelin, bacillibactin, paenibactin, myxochelin, griseobactin, benarthin, tailcyclized, and samylocyclicin.

**Conclusion:**

The strain TA-1 had a significant biological control effect against peanut early leaf spot disease *in-vitro* and in greenhouse assays. Whole genome analysis revealed that, TA-1 strain belongs to *B. amyloliquefaciens* and could produce the antifungal secondary metabolites.

**Research**.

## Background

Fungal pathogens have the potential to significantly reduce agricultural productivity and pose a significant threat to global food security [1, 2]. Fungi are predominantly present on the foliage of plants; however, they can also impact other anatomical structures of the plant [3]. In the case of peanut plants, the leaves of the plant were the primary target of the fungus [4]. A fungus known as *C. arachidicola* causes early leaf spot disease in peanuts [5]. As one of the most significant groups of plant pathogenic fungi that cause leaf spots, *cercosporoids* cannot be ignored. These diseases affect dicots, monocots, gymnosperms, and ferns of almost every continent (including cultivated plants) [6, 7].

The application of agrochemicals is employed to sustain crop yield and mitigate the impact of plant pathogens, which can potentially compromise ecological and human health [8, 9]. These agrochemicals can be minimized by cultivating resistant varieties and using biocontrol agents. As a result of these approaches, the environment will be protected and ecological balance will be sustained [10]. However, a lack of disease-resistant germplasm and the practice of continuous cropping make it difficult to control pathogens, which hinders the cultivation of resistant varieties [11]. Utilizing biocontrol microorganisms to mitigate plant pathogens and insects is a more effective approach [12]. Rhizosphere bacteria may produce biological pesticides that combat plant diseases and create systemic resistance [9, 10]. Specifically, Rhizospheric bacteria belonging to the *Bacillaceae* family have been discovered to contain bioactive molecules that exhibit growth-promoting and antagonistic effects against plant pathogens [13, 14]. Bacillus is a widely used biocontrol agent due to its rapid growth rate, ability to withstand unfavorable environmental conditions, and acid resistance, which allows for storage at low temperatures [15, 16].

Recent research has indicated that the utilization of bacillus species can positively impact the growth of tomato, cotton, cucumber, tobacco, and lettuce crops, as well as disease control. These species included *B. subtilis*, *B. amyloliquefaciens*, *B. brevis*, and *B. cereus* [17, 18]. It was demonstrated that The *B. amyloliquefaciens* strain exhibits efficient antagonistic effects, enabling it to effectively control disease outbreaks and induce plant resistance against such diseases [19]. Moreover, *B. amyloliquefaciens* strains have strong antimicrobial properties due to the non-ribosomally synthesized lipopeptides they produce (e.g., surfactin, iturin, and fengycin) [20]. As a result, *Bacillus* species are effective agents for biocontrol [21]. A comprehensive study has been conducted worldwide, including in China, to evaluate the potential of *Bacillus* as a biological control agent [22]. At the moment, China has registered 110 products that can be used for plant disease management and insect pest control (China Pesticide Information Network. http://www.chinapesticide.org.cn/). However, there have been no reports of *Bacillus* species being used to control peanut early leaf spot disease.

Omics approaches were utilized by researchers to identify genetic components implicated in plant growth promotion, secondary metabolite production, and beneficial microorganism habitat adaptation [23–25]. The utilization of third-generation sequencing techniques, such as whole-genome sequencing (WGS), may instantly, cost-effectively, and effectively generate a complete bacterial genome sequence [26]. By using WGS and online databases, such as GO, KEGG, COG, and NR, it is possible to identify differences between species within the same genus. Various microbial communities, including those in intestinal flora, soil, and fungi, can be identified using WGS [27]. Several bioinformatic programs (‘ClustScan,‘ ‘CLUSEAN,‘ ‘antiSMASH,‘ ‘SMURF,‘ ‘MIDDAS-M,‘ ‘ClusterFinder,‘ ‘CASSIS/SMIPS,‘ and ‘C-Hunter’) are used to assist with whole-genome analyses to find the clusters of genes that are responsible for synthesizing antibiotics and other secondary metabolites [28, 29].

Based on the above facts, our study aimed to unravel the genome complexity of *B. amyloliquefaciens* TA-1 to identify the genetic factors underlying its biocontrol and plant growth promoting properties. WGS, combined with a detailed bioinformatics analysis, identified novel gene clusters in strain TA-1 that encoded for CAZymes and secondary metabolites. This study provides insight into the genome of *B. amyloliquefaciens* TA-1 and thus exploits its genetic potential in future research.

## Materials and methods

### Microorganism and culturing conditions

Both the pathogenic and antagonistic strains were obtained from the Institute of Plant Protection, Liaoning Academy of Agricultural Sciences, China. The antagonistic bacterial strain TA-1 was cultured in LB agar medium containing (10 g/L tryptone, 5 g/L yeast extract, 5 g/L NaCl, and 15 g agar/L) for a duration of 3 days at a temperature of 28℃ under dark conditions. Culturing of the pathogenic fungus *C. arachidicola* was carried out on potato dextrose agar (PDA) medium containing (250 g/L potato, 20 g/L glucose, and 15 g/L agar). The incubation was performed for a period of 14 days at a temperature of 28℃ under dark conditions.

### Mode of submerged fermentation

The two-stage submerged fermentation process was carried out. To produce the spore suspension, a 250 mL flask comprising 40 mL of LB medium was inoculated with two 5-mm spore cakes of TA-1 strain. Subsequently, the fermentation medium was formulated with precise measurements of 2.5 g/L of sodium chloride, 10 g/L of tryptone, and 7 g/L of yeast extract. The pH was then meticulously adjusted from 6.6 to 6.8. Subsequently, the fermentation medium underwent sterilization via autoclaving. Aseptic inoculation was performed by introducing a spore culture of strain TA-1 at a concentration of 5% (v/v) into a 250 mL flask containing 45 mL of fermentation media. The fermentation medium was subjected to incubation in a rotary shaker at a temperature of 30℃ and a shaking rate of 165 rpm for a period of 96 h.

### In vitro study; antagonistic effects of strain TA-1 against *C. arachidicola*

The antagonistic potential of strain TA-1 against *C. arachidicola* was assessed using the Oxford cup method. The pure fermentation broth was used as a treatment, whereas two other dilution concentrations (10% and 20%) were prepared by adding distilled water to the pure fermentation broth. Pour 18 mL of molten potato dextrose agar (PDA) into a 90 mm petri dish. Afterward, a 5 mL spore suspension of *C. arachidicola* was introduced, which was prepared in potato dextrose broth (PDB). A sterile Oxford cup was aseptically positioned at the center of the sterile petri dish. Using a sterile ependorf tube, 200μL of the fermentation broth was aseptically inoculated into the Oxford cup. The control group was subjected to tebuconazole fungicidal treatment. The treatment dosage was the same as in the treatment groups. For the negative control comparison, there was no treatment in the Ck group, only inoculated with *C. arachidicola* (CA), while in the other treatment, it was inoculated with CA and then treated with pure fermentation broth of strain TA-1. Later on, we incubated it in a dark growth chamber at a temperature of 28 °C for 14 days. The antagonistic potential of TA-1 was assessed by measuring the inhibition zone of *C. arachidicola*.

### Effects of strain TA-1 on mycelial morphology and hyphal ultrastructure of *C. arachidicola*

We used the Oxford cup technique to investigate the impact of strain TA-1 on mycelial morphology and hyphal ultrastructure of *C. arachidicola*. A 5 mL spore suspension of *C. arachidicola* was aseptically transferred onto the surface of each Petri plate, as described in the above section in vitro study. Subsequently, the pure fermentation broth was used as the treatment agent, while the untreated sample acted as the control (CK).We chose hyphae that grew near the edge of the inhibition zone so we could study the mechanism by which the TA-1 strain affected the *C. arachidicola*. A scanning electron microscope (SEM) (Hitachi) was used to explore morphological alterations.

### Disease control effects in green house

Two peanut seeds were sown in each 4-inch plastic pot containing nutrient-rich, hygienic soil and kept in green house environment. The plants had free access to fresh air, sunshine, and humidity at a temperature of 30 to 24 °C. After the plants had grown for 30 days, they were given the treatments. The five-leaf (20 leaflets) were inoculated by drenching with 10 ml of inoculum of *C. arachidicola* (CA), 10 ml of TA-1 broth mixed with 10 ml of inoculum of *C. arachidicola* (CA + TA-1), and 10 ml of fungicide (tebuconazole) mixed with 10 ml of inoculum of *C. arachidicola* (CK), respectively. To calculate the disease incidence and disease control efficiency, we established the following formula:

$$Disease\,incidence\,\% = \frac{{Total\,leaflets - Infected\,lealets}}{{Total\,leaflets}} \times 100$$

### Whole-genome sequencing, assembly, and functional annotation

The genomic DNA of strain TA-1 was isolated using the Rapid Bacterial Genomic DNA Isolation Kit (Sangon Biotech Co., Ltd.) in accordance with the manufacturer’s instructions. The Illumina HiSeq sequencing platform was utilized to perform whole-genome sequencing. The resulting data files were subjected to CASAVA base calling analysis to obtain raw sequences, which were then stored in FASTQ file format. The sequencing data was filtered using Trimmomatic version 0.36 to produce high-quality data, and the quality, reads, trimming, and de novo assembly were visually checked using FastQC version 0.11.2. SPAdes 3.5.0 was used to compile the next-generation sequencing data. The spliced contigs were complemented with GapFiller v. 1.11, while splicing errors and minor insertions and deletions were corrected using PrInSeS-G v. 1.0.0. Prokka v. 1.10 was utilized to predict the gene elements of the assembled data. Prodigal was used to find the coding genes. Aragorn was used to find the tRNAs, RNAmmer was used to find the rRNAs, and Infernal was used to find the miscRNAs. For the 16s RNA genome study of 30 typical *Bacillus* strains with sequenced genomes, a phylogenetic tree was built using the FAST Tree program with default parameters.

The pan-genome report of 25 common *Bacillus* strains with sequenced genomes was used to generate a phylogenetic tree using the bacterial pan genome analysis pipeline (PGAP) software with its default parameters [30]. The protein sequences were aligned with those in the Pathogen-Host Interactions Database (PHIbase)^1^, the Clusters of Orthologous Groups of Proteins (COG)^2^ database, the Conserved Domain Database (CDD)^3^, the NCBI non-redundant protein sequences (NR)^4^ database, and the Protein Family (Pfam)^5^ database using Blast + v2.2.28 from the National Center for Biotechnology Information. Gene Ontology (GO)^6^ analysis was done utilizing protein annotation data from the Swiss-Prot and TrEMBL databases, as well as annotation information from the (UniProt database)^7^. A pathway enrichment analysis was performed using the Kyoto Encyclopedia of Genes and Genomes (KEGG)^8^ Automatic Annotation Server [31]. The gene set protein sequences were aligned with the Carbohydrate-Active enZYmes database (CAZy)^9^ using HMMER3 v. 3.1b1, yielding the relevant carbohydrateactive enzyme annotation information (E-value 10.5).

### Identification of secondary metabolite gene clusters

The secondary metabolite synthesis gene clusters of *Bacillus amyloliquefaciens* TA-1 were discovered with antiSMASH 6.0 [32].

### Statistical analysis

The data was analyzed using one-way ANOVA, and Tukey’s HSD was performed to evaluate for significance at p > 0.05. IBM SPSS version 25.0 was used for the statistical analysis. The means that shared the same letters were statistically nonsignificant. The superscripts a, b, and c show if the pairwise comparisons are statistically different. To analyze the data, all treatments were performed three times.

## Results

### In vitro study; antagonistic effects of strain TA-1 against *C. arachidicola*

**Fig. 1:**
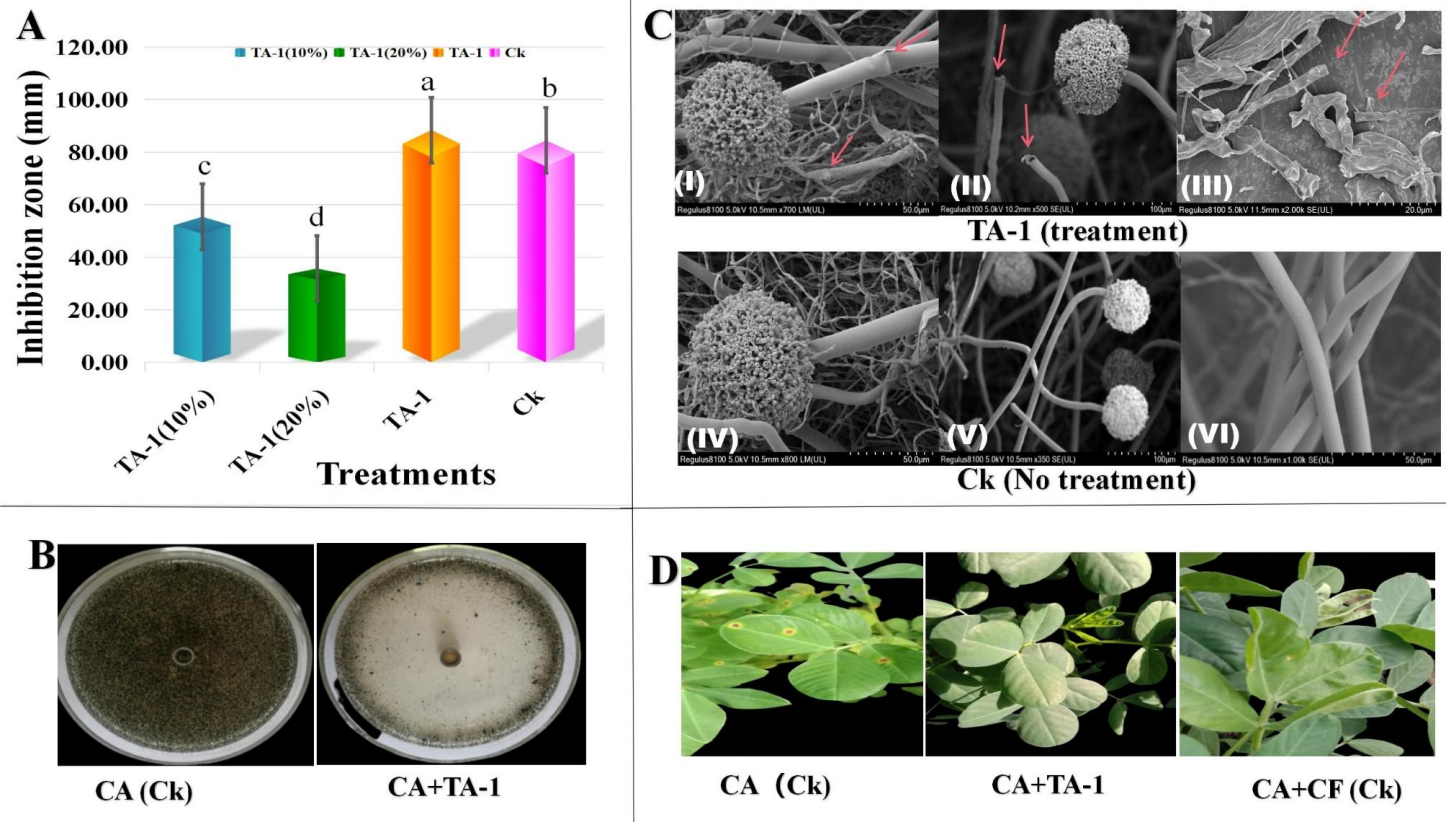
In-vitro antifungal activity of the TA-1 strain at different concentrations against *C. arachidicola.* Ck (chemical fungicide) (**A**). An illustration of the negative control effects, CA (Ck), only inoculated with *C. arachidicola)*, CA + TA-1 inoculated with *C. arachidicola* and treatment with TA-1 (**B**). SEM images of hyphal morphology of *C. arachidicola* after the treatment of TA-1 (up side, I, II, III), the arrows showing the deformations in the ultra-structure of mycelium of *C. arachidicola* and in Ck (control group), no treatment (down side, IV, V, VI) (**C**). An illustration of disease control efficiency on peanut early leaf spot, CA (Ck); only inoculated with *C. arachidicola*, CA + TA-1; inoculated with *C. arachidicola* + treated with TA-1, and CA + CF (Ck); inoculated with *C. arachidicola* + treated with chemical fungicide (**D**)

The strain TA-1 displayed strong antagonistic effects using the Oxford cup method. The inhibition activity of different dilutions against *C. arachidicola* was impressive; specifically, pure broth had a significant inhibition zone of 88.39 mm, while 10 and 20% diluted broths had lesser activity. On the other hand, the control group also (CK) displayed a strong inhibition zone of 84.45% (Fig. 1A). Figure 1B shows an illustrative representation of the negative control. The results of the study revealed that the growth of *C. arachidicola* was observed to be optimal without any treatment in the control group (Ck). On the other hand, the application of the TA-1 strain had a strong inhibition zone against *C. arachidicola*.

### Effects of strain TA-1 on mycelial morphology and hyphal ultrastructure of *C. arachidicola*

The SEM images made it clear that the *C. arachidicola* hyphae were broken and malformed. The hyphae treated with TA-1 shown morphological alterations, which resulted in a damaged and broken appearance, along with evident indications of significant damage. The red arrows indicated the regions of impact on the fungal mycelium, as shown in Fig. 1C, notably in the upper part, denoted as I, II, and III. In comparison, the hyphae of untreated *C. arachidicola* showed a smooth and rounded morphology, as clearly shown in Fig. 1C within the lower part of the figure and denoted as IV, V, and VI.

### Disease control effects in green house

The disease control efficiency of the strain TA-1 was 95% when peanut plant leaves were inoculated with *C. arachidicola* (CA) and then treated with pure fermentation broth of the strain TA-1. In the positive control group, when peanut plant leaves were inoculated with CA and then treated with a chemical fungicide, the disease control efficiency was also 95% (Table 1).

**Table 1:** Green house assay, disease incidence, and disease control effects of the TA-1 strain

Treatment group	Disease incidence	Disease control%
Inoculated with *C. arachidicola* (CK)	90%	-
Inoculated with *C. arachidicola* + Strain TA−1	5%	95%
Inoculated with *C. arachidicola*+Tebuconazole (CK)	5%	95%

- indicating, no disease incidence or no disease control

The disease incidence of the negative control group (Ck) inoculated only with CA was 90%, while the disease incidence of the treatment group, where the peanuts leaf was inoculated with CA and then treated with TA-1 was 5%. In the same way, in positive control, where peanut leaves were inoculated with CA, and then treated with chemical fungicide also had 5% disease incidence, as shown in the (Table 1). Figure 1D shows an illustrative representation of the disease control impacts exhibited by the strain TA-1 when compared with the positive and negative controls. The results showed that peanut plants that were treated with the negative control CA had an abundance of leaf spot disease, while plants that were treated with CA + TA-1 didn’t seem to have any leaf spot disease. In the same way, there didn’t seem to be any leaf spot disease in the positive control group when a chemical fungicide, CA + CF, was used.

### Whole-genome sequencing, assembly, and functional annotation

**Fig. 2:**
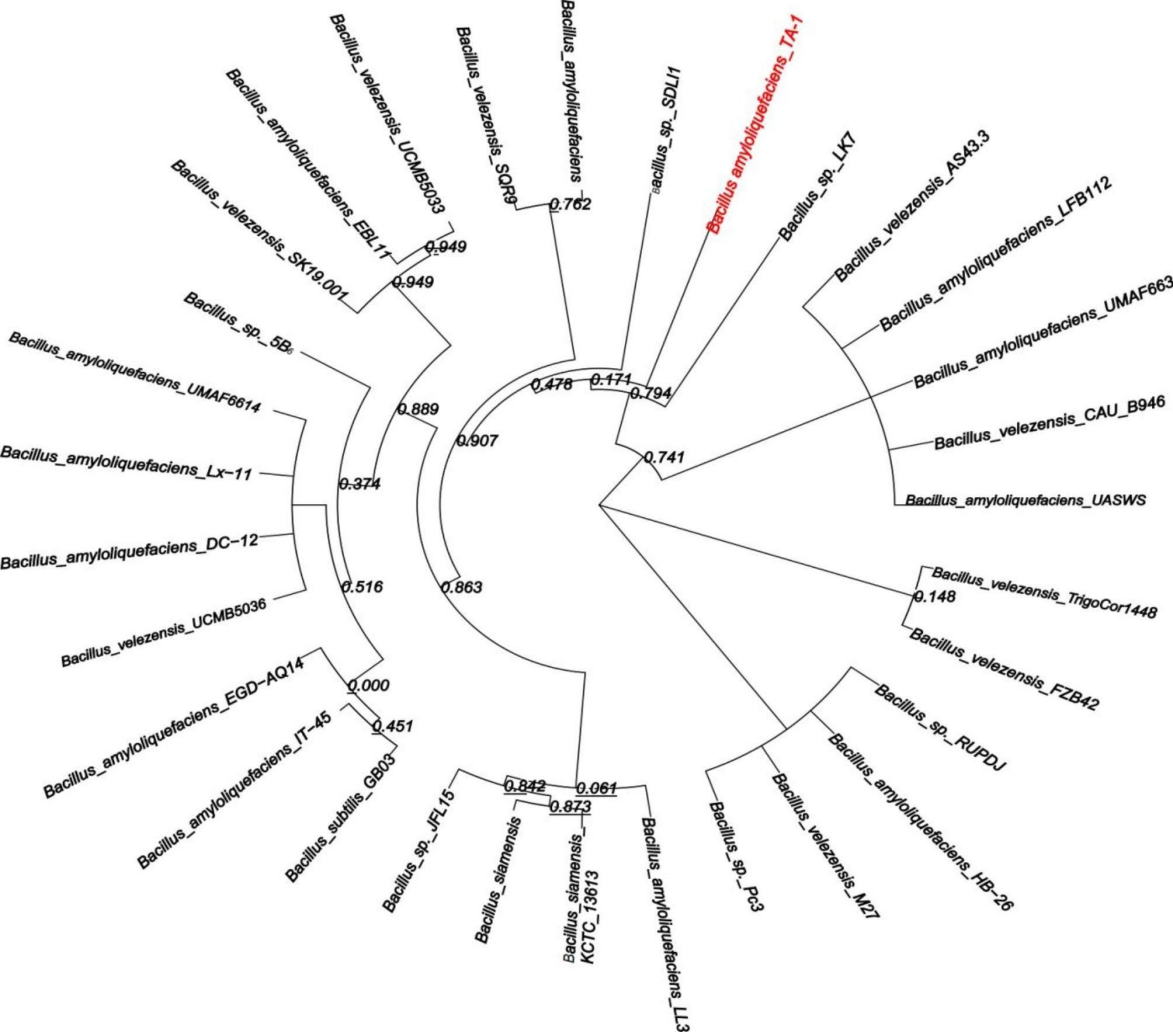
Phylogenetic tree based on 16 S rRNA

**Fig. 3:**
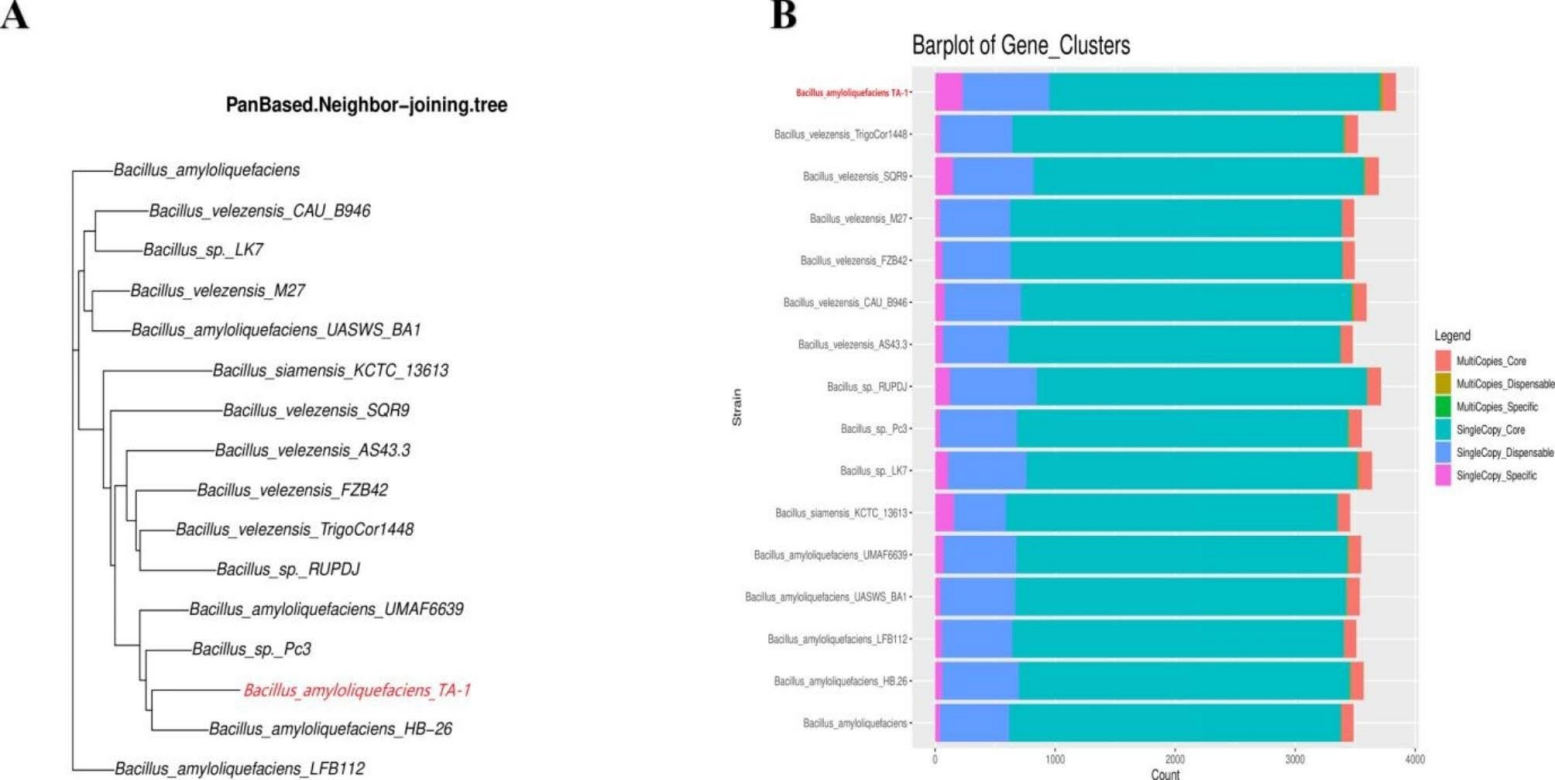
Phylogenetic tree based on pan genomes (**A**). Genome-wide proportions of core, accessory, unique, and exclusively absent genes were analysed among 30 *Bacillus* species. (**B**)

**Fig. 4:**
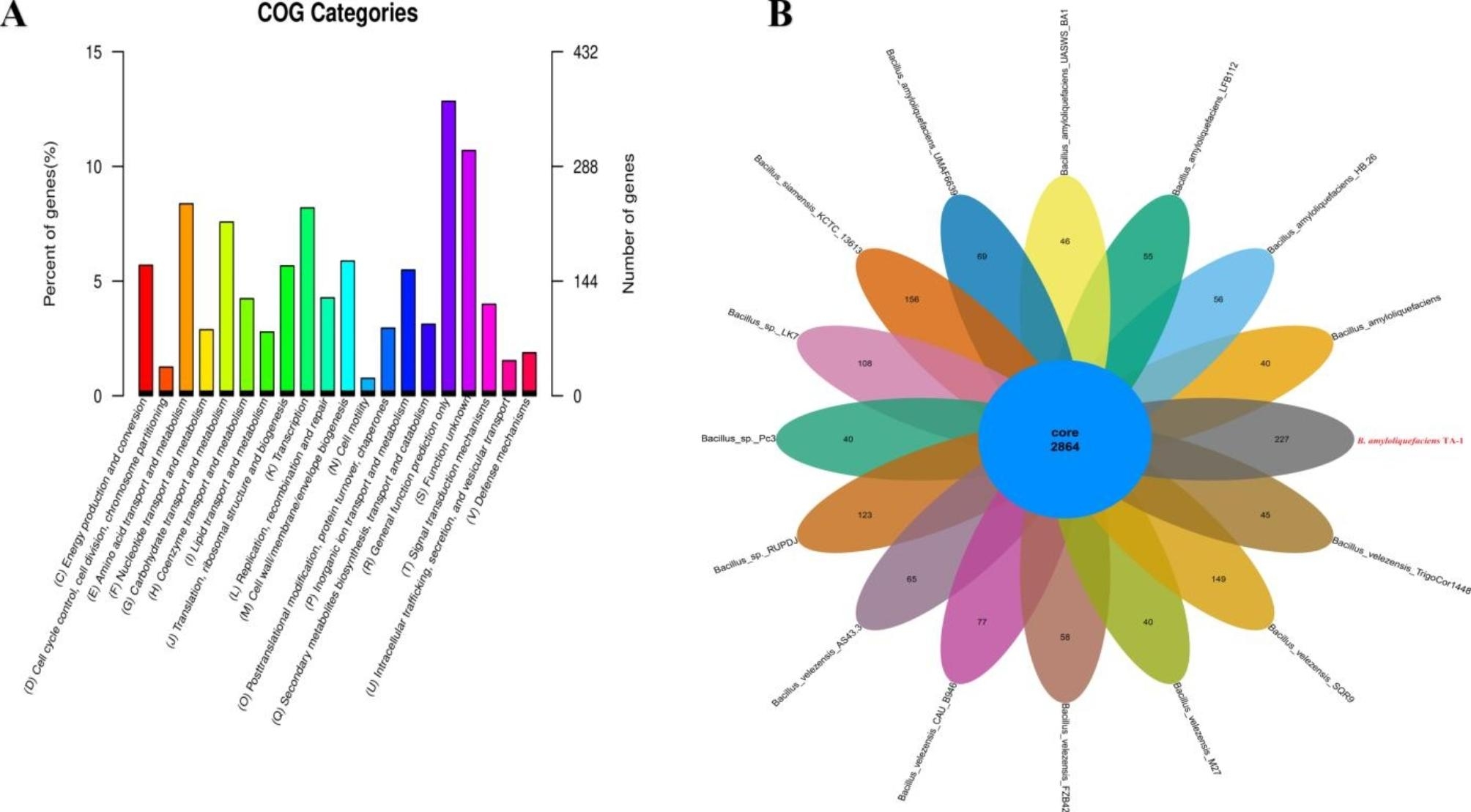
Displayed in order from outside to inside: scale bar, GC content (GC: ratio of guanine and cytosine), sequencing depth, gene element display, and clusters of orthologous group functional display (**A**). Flower plot showing numbers of species-specific genes commonly found in each genome of each species (in the petals), and TA-1 core orthologous gene number (in the center) (**B**)

Based on 16s RNA, the phylogenetic analysis showed that strain TA-1 was clearly clustered with *B. amyloliquefaciens* and different from other *Bacillus species* (Fig. 2). Based on pan genome data, the phylogenetic analysis showed that strain TA-1 was clearly clustered with *B. amyloliquefaciens* and different from other *Bacillus* species (Fig. 3A). Similar number of core genes were present in the genomes of strain TA-1 and 15 other *Bacillus* strains, however, the proportions of accessory, peculiar, and strain-specific genes were only found in the TA-1 strain (Fig. 3B). In the end, the strain TA-1 genome was combined into 17 contigs. These contigs had a total length of 4,135,865 base pairs (the N50 contig size was around 1,035,320 base pairs), a guanine and cytosine (GC) content of 45.91%, 4172 protein-coding genes, 81 tRNAs, 9 rRNAs, and 90 unknown genes (Table 2). Twenty functional groups were created from a total of 2881 annotations from the COG database, with the majority of these groups being concerned with transcription, secondary metabolite biosynthesis, amino acid transport and metabolism, and carbohydrate transport and metabolism (Fig. 4A). In Orthologus cluster analysis, a flower plot showed the number of common and unique genes. The results indicated that the strain TA-1 contained 2864 common genes, while 227 genes were unique (Fig. 4B).

**Table 2:** Genome features of strainTA-1

Features	Genomes
Genome size Bp	4,135,865
Gene Numbers	4172
Gene total length	4,135,865
G + C content %	45.91
Genome coverage	89.9
Contings	17
Contings N50 (bp)	1,035,320
tRNA genes	81
rRNA genes	9
Protein coding genes	4172
Genome accession number	JARDRQ000000000

**Fig. 5:**
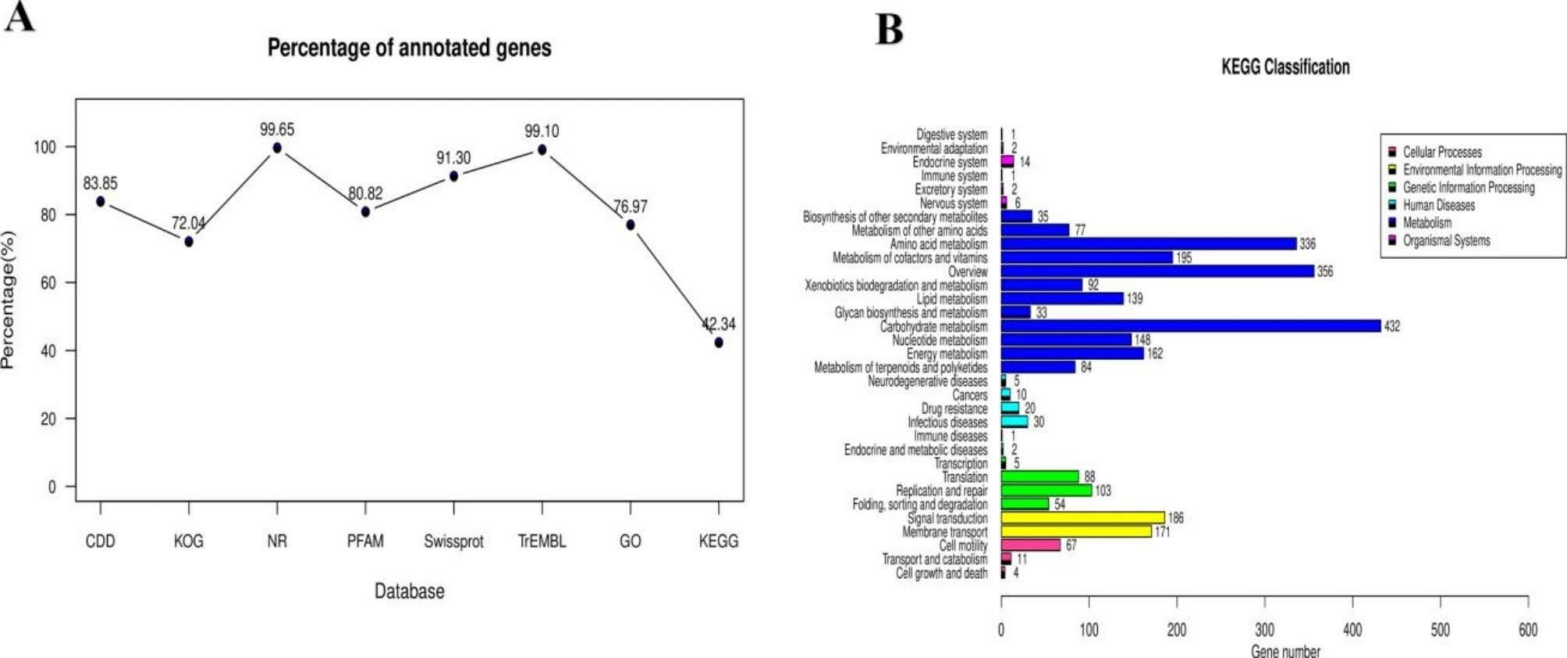
Functional analysis of gene and protein sequence annotations from strain TA-1. Proportions of genes annotated in eight databases (**A**). Metabolic pathways annotated using the Kyoto Encyclopedia of Genes and Genomes (KEGG) database (**B**)

The functional annotation of the whole genome of strain TA-1 was performed utilizing eight different gene annotation databases (Fig. 5A). There were a total of 3985 annotations discovered in the NR database, which represents 99.65% of the total number of annotations. In addition, the Swiss-Prot, CDD, Pfam, and TrEMBL databases, in that order, had 3651 (91.30%), 3353 (83.85%), and 3232 (80.82%), whereas the TrEMBL database contained 3963 (99.10%) annotations. There were a total of 1693 KEGG annotations that were connected to 33 different metabolic pathways. The genome of TA-1 has the largest number of genes linked with metabolism (2087), followed by carbohydrate, amino acid, and cofactor metabolism, vitamin metabolism, and processing of environmental information (357), processing of genetic information (253), and cell processes (82) (Fig. 5B).

**Fig. 6:**
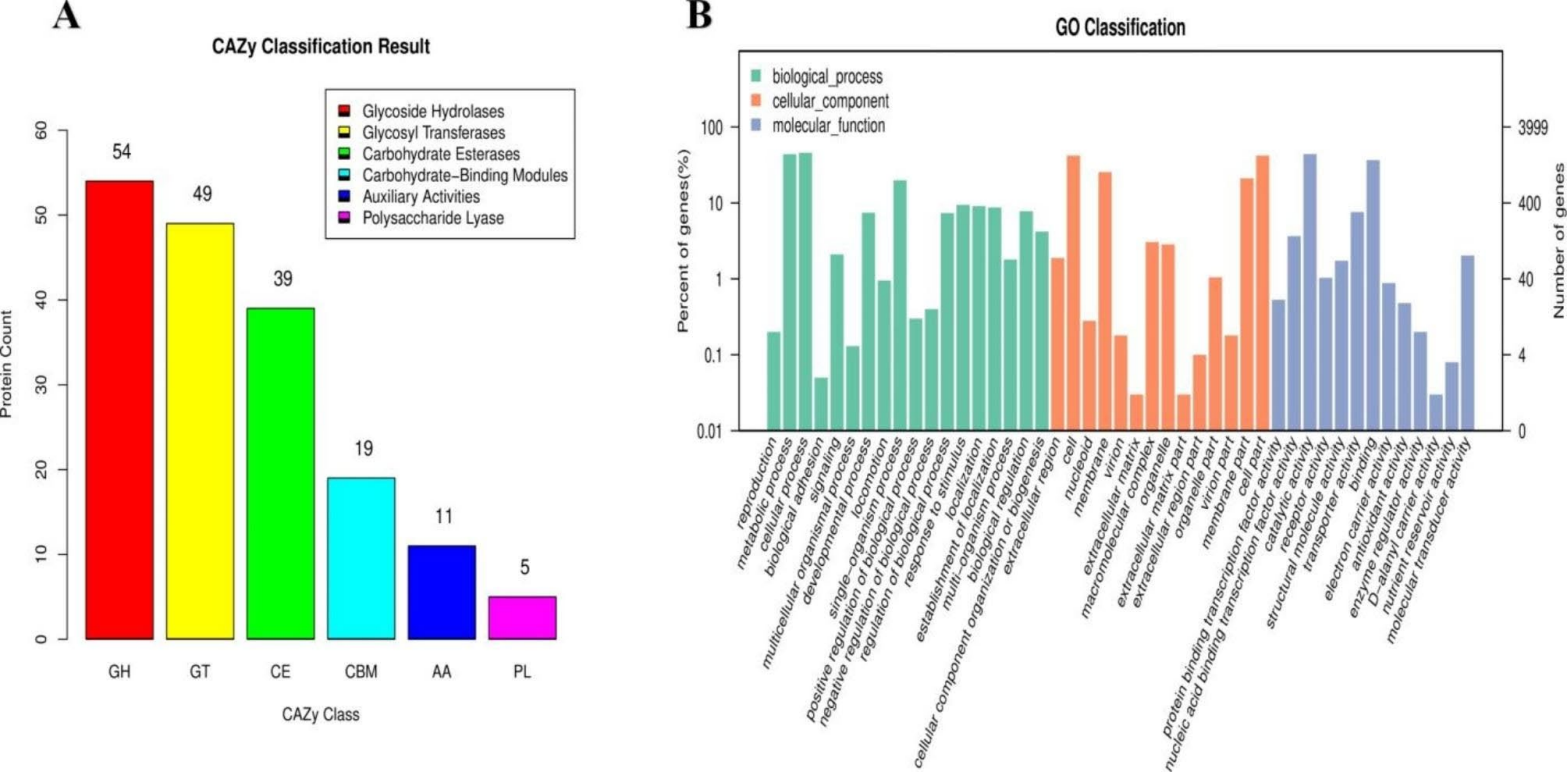
Functional analysis of gene and protein sequence annotations from strain TA-1. Proportions of enzymes annotated using the Carbohydrate-Active enZYmes database (**A**). Biological process, cellular component, and molecular function terms annotated using the Gene Ontology database (**B**)

The CAZy database contained a total of 177 annotations, which included glycoside hydrolases (54), glycosyl transferases (49), carbohydrate esterases (39), auxiliary activities (11), carbohydrate-binding modules (19), and polysaccharide lyases. Glycoside hydrolases accounted for 54 of the annotations, glycosyl transferases for 49, and carbohydrate esterases for 39. (5). (Fig. 6A) The distribution of CAZymes as a whole showed that glycosyl transferases, glycoside hydrolases, and carbohydrate esterase-related enzymes are all involved in the non-ribosomal production of secondary metabolites. An analysis of the gene ontology showed that there were 18 GO terms that belonged to biological processes, 14 GO terms that belonged to cellular processes, molecular functions of TA-1 that were connected to catalytic activity, and 13 GO terms that belonged to molecular functions. The GO analysis found that the most important molecular activities were TA-1 functions, while the most important cellular components were cell and membrane components (Fig. 6B).

### Secondary metabolites synthesis gene clusters

**Fig. 7:**
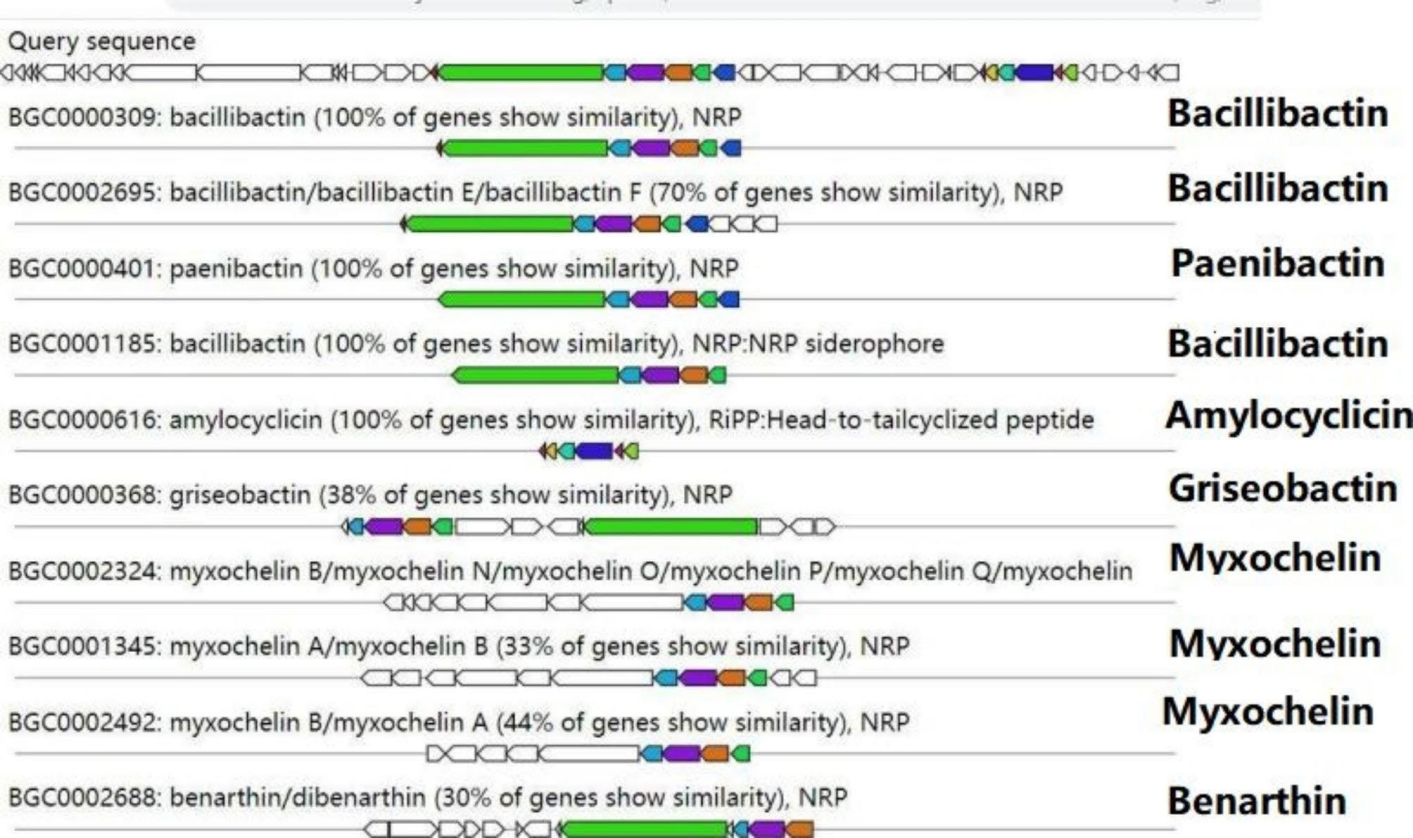
Secondary metabolites’ gene clusters with antimicrobial metabolites in strain TA-1, identified by antiSMASH 6.0

It was found that the genome of strain TA-1 has genes that code for new secondary metabolites that suppress fungi (Fig. 7). The strain TA-1 genome has seven gene clusters that express NRPS (non-ribosomal peptide synthetase), one gene cluster that codes for the biosynthesis of siderophores, one gene cluster that codes for the biosynthesis of tailcyclized peptides, and one gene cluster that codes for the biosynthesis of myxochelin. One of the seven gene clusters encoding for NRPS showed 100% similarity to genes involved in bacillibactin, while another showed 70% similarity to genes involved in bacillibactin. Another cluster shared all of the genes involved in making paenibactin. The two clusters shared, respectively, 44% and 33% of the genes involved in making myxochelin.It was found that the other two NRPS-encoding clusters shared 38% and 30% of their genes with genes that make griseobactin and benarthin, respectively. The gene cluster encoding siderophore production was found to be 100% identical to bacillibactin. Similarly, the gene cluster encoding tailcyclized peptide synthesis was shown to be 100% identical to amylocyclicin.

## Discussion

This study investigated the biological control of *C. arachidicola* to suppress the peanut leaf spot by the antagonist strain *B. amyloliquefaciens* TA-1. Peanut leaf spot affects the peanut harvest, quality, and economy. The management of leaf spots is crucial for preventing defoliation and yield losses. According to Anco et al., 2020 [33], leaf spots in peanuts resulted in defoliation, which reduced yields by 40–50%. Results revealed that, the in vitro antagonistic activity of the TA-1 strain against *C. arachidicola* was significant. There is no doubt that *B. amyloliquefaciens* is one of the most effective biological control agents [34]. The results of the pot experiments also revealed the strong potency of TA-1, as the disease incidence was very low and the disease control efficiency was very high. So, both the in vitro and green house experiments indicated that the TA-1 strain could be a potent biocontrol agent against *C. arachidicola*. *Bacillus* strains were evaluated on various crops and found to be effective against a number of fungal plant pathogens and diseases, such as *Fusarium wilt* in tomatoes [35], FHB in wheat [36], and barley [37]. Scanning electron microscopy analysis indicated that the hyphae of *C. arachidicola* were broken and severely damaged by the effects of the TA-1 strain. Zalila et al., 2016 [36] reported that, *Bacillus*-derived compounds, can disrupt the morphology and break the cell wall of the fungus. This study used whole-genome sequencing and comparative genomics to investigate the molecular processes and antifungal genes of strain TA-1, which could be used as a biocontrol agent. Strain TA-1 was confirmed as *Bacillus amyloliquefaciens* based on 16 S rRNA, pan and core genome analyses. The 16 S rRNA gene has been widely used for strain identification, but microbial taxonomies based on it have low phylogenetic resolution. In recent times, the formation of phylogenies through the utilization of the core genome has made progress toward the formation of a standardized taxonomy for bacteria [38]. The flower diagram of orthologous clusters revealed that strain TA-1 shared a number of unique genes with homologous gene clusters. The fields of whole-genome sequencing and comparative genomics are making it easier to look at bacterial molecular processes and antifungal genes as possible biocontrol agents [39].

The application of de novo whole-genome sequencing in this investigation revealed the key role of secondary metabolites in the antifungal mechanisms of strain TA-1. *Bacillus amyloliquefaciens* is recognized for its potential to synthesize a range of metabolites having antimicrobial properties [40]. Annotations of gene and protein sequences showed that several carbon, amino acid, and energy metabolism pathways were involved in iron uptake and metabolism, movement and chemotaxis, membrane transport, and other good traits. Most of the carbohydrate-active enzymes that have been found are glycoside hydrolases, glycosyl transferases, and carbohydrate esterases. These enzymes help make secondary metabolites through non-ribosomal pathways [39]. Secondary metabolite gene clusters in strain TA-1 were discovered, as well as various predicted siderophores, tailcyclized peptides, myxochelin, bacillibactin, paenibactin, myxochelin, griseobactin, benarthin, tailcyclized peptides, and amylocyclicin. Recently, it was reported that the *B. amyloliquefaciens subsp. plantarum* strain Fito_F321 had thirteen clusters of genes for the synthesis of secondary metabolites [41]. The relationship between putative secondary metabolites and their related biosynthetic genetic clusters is made possible by genome mining [42, 43].

Majority of the secondary metabolites present in TA-1 strain are non-ribosomal peptide synthetase (NRPS). Crude lipopeptide extracts of *B. amyloliquefaciens* SS-12.6 inhibited the severity of sugar beet leaf spot disease [44], while the FZB42 strain controlled *F. graminearum* [36]. The presence of a siderophore indicated that the strain TA-1 also had the ability to promote plant growth. It is reported that siderophore is involved in plant growth promotion [45]. Bacillibactin is a kind of iron chelator that has the potential to bind soluble iron ions, which are required for the activity and proliferation of pathogens [46]. The study findings concluded that strain TA-1 was a *Bacillus amyloliquefaciens* bacterium with potent antagonistic potential against *C. arachidicola in-vitro* and *in-vivo*, which could be a potent biological control agent of peanut early leaf spot. *B. amyloliquefaciens* has not been used to control peanut early leaf spot as a possible biocontrol microbial resource. Moreover, several gene clusters were found that can produce the antifungal secondary metabolites.

## Conclusion

In this study, strain *B. amyloliquefaciens* TA-1 inhibited the growth of the pathogen *C. arachidicola* that causes early leaf spot. In-vitro experiments revealed that *B. amyloliquefaciens* TA-1 showed broad-spectrum antifungal action. The antagonistic effects of TA-1 distorted and damaged the ultrastructure and hyphea of *C. arachidicola*. Experiments in a greenhouse also revealed that the *B. amyloliquefaciens* TA-1 strain was a potent biocontrol agent against the peanut early leaf spot disease. De novo whole-genome sequencing found the significant traits that led to TA-1’s antagonistic effects and predicted the production of biologically active compounds like siderophore, tailcyclized peptide, myxochelin, bacillibactin, paenibactin, myxochelin, griseobactin, benarthin, tailcyclized, and amylocyclicin. The study’s findings may be beneficial for the production of biocontrol products for peanut early leaf spot disease control and management.

## Data Availability

The wholegenome sequence was submitted to NCBI under GenBank accession number: JARDRQ000000000. Bioproject ID:PRJNA937395.

## Footnotes

1 PHI-base: http://www.phi-base.org/.

2 NCBI COG/KOG: https://www.ncbi.nlm.nih.gov/COG/.

3 CDD: https://www.ncbi.nlm.nih.gov/cdd/.

4 NCBI NR: http://ncbi.nlm.nih.gov/.

5 PFAM: http://pfam.xfam.org/.

6 GO: http://www.geeontology.org.

7 Uniprot: http://www.uniprot.org.

8 KEGG: http://www.kegg.jp.

9 CAZy: http://www.cazy.org/.
